# Enhanced Electrochemical Properties of Non-stoichiometric Layered Perovskites, Sm_1−x_BaCo_2_O_5+d_, for IT-SOFC Cathodes

**DOI:** 10.3389/fchem.2021.633868

**Published:** 2021-04-21

**Authors:** Chan Gyu Kim, Sung Hun Woo, Kyeong Eun Song, Seung-Wook Baek, Hyunil Kang, Won Seok Choi, Jung Hyun Kim

**Affiliations:** ^1^Department of Advanced Materials Science and Engineering, Hanbat National University, Daejeon, South Korea; ^2^Interdisciplinary Materials Measurement Institute, Korea Research Institute of Standards and Science (KRISS), Daejeon, South Korea; ^3^Department of Electrical Engineering, Hanbat National University, Daejeon, South Korea

**Keywords:** layered perovskite, cathode, intermediate temperature-operating solid oxide fuel cell, non-stoichiometric composition, area specific resistance, electrical conductivity

## Abstract

In this study, electrochemical properties of layered perovskites having non-stoichiometric compositions (Sm_1−x_BaCo_2_O_5+d_, *x* = 0, 0. 01, 0.02, 0.03, 0.04, 0.05, 0.10, and 0.15) were analyzed for the direct application of cathode materials for Intermediate Temperature-operating Solid Oxide Fuel Cells (IT-SOFC). From the Sm_1−x_BaCo_2_O_5+d_ oxide systems calcined at 1,100°C for 8 h, single phase (SmBaCo_2_O_5+d_, SBCO_1) was maintained only in the case of the *x* = 0 composition. In the compositions of *x* = 0.05–0.10, BaCoO_2.6_ was mixed with the pattern of SBCO. In addition, in the composition of *x* = 0.15, it was confirmed that BaCoO_2.6_ and CoO phases coexisted with SBCO. In the compositions of Sm_1−x_BaCo_2_O_5+d_, the overall Area Specific Resistance (ASR) values decreased as the removal amount of Sm increased from *x* = 0–0.10; then, the values increased for compositions from *x* = 0.15. For example, the ASRs of SBCO_1, Sm_0.95_BaCo_2_O_5+d_ (SBCO_0.95), Sm_0.90_BaCo_2_O_5+d_ (SBCO_0.90), and Sm_0.85_BaCo_2_O_5+d_ (SBCO_0.85) measured at 600°C were 0.301, 0.147, 0.119, and 0.179 Ω cm^2^, respectively. In particular, SBCO_0.90 was found to have an excellent ASR property of about 0.035 Ω cm^2^ at 700°C. Typical properties of the metal–insulator transition (MIT) electrical conductivity were shown in all measured compositions. The temperature at which MIT occurred increased as the non-stoichiometric composition increased.

## Highlights

- Sm_1−x_BaCo_2_O_5+d_ (*x* = 0, 0.01, 0.02, 0.03, 0.04, 0.05, 0.10, and 0.15) oxide systems having non-stoichiometric compositions were synthesized by solid state reaction.
- BaCoO_2.6_ phase was identified from the composition of *x* = 0.05 or more in Sm_1−x_BaCo_2_O_5+d_; CoO phase was also found to exist for the composition of *x* = 0.15
- All composition showed higher electrical conductivity values than 100 S/cm in the temperature ranges of 500–750°C.
- Area specific resistance (ASR) of Sm_1−x_BaCo_2_O_5+d_ was 0.15 Ω·cm^2^ or less at 650°C. SBCO_0.90 showed the lowest value of 0.119 Ω·cm^2^ at 600°C

## Introduction

As environmental problems such as global warming, caused by the indiscriminate use of fossil fuels since the beginning of the industrial revolution in the 19th century have recently emerged, interest in eco-friendly energy has been expanding (Wigley, 1998; Hoad, 2015). Accordingly, many countries around the world have been implementing nature-friendly policies and spurring green energy development. There are various nature-friendly energy generation methods, including wind, hydroelectric, and solar power However, these power generation systems have disadvantages such as restrictions on installation locations and high initial cost. Therefore, there is need for development of other energy methods that can replace these systems and mitigate their problems (Rodman and Meentemeyer, 2006; Kosnik, 2008; Ingole and Rakhonde, 2015).

Fuel cells or fuel cell systems based on hydrogen energy have been emerging as alternative energy conversion methods to replace the energy generating from fossil fuel. Especially, among various fuel cell types, solid oxide fuel cells (SOFC) are the focus of attention recently. An SOFC is an energy converting device that produces water and generates electricity via the chemical reaction of hydrogen and oxygen (Minh, 2004).

SOFCs are known to show higher values of energy efficiency and power density than those of other fuel cells. SOFCs, which are made of ceramics, are composed of a dense electrolyte and two porous electrodes (cathode and anode). The SOFC structure is simpler than those of other types of fuel cell. In addition, due to their high exchange current density and the kinetics characteristics that occur under relatively high temperature operating conditions, SOFCs do not require any precious or expensive catalysts for operation.

While SOFCs have advantages of high efficiency at high temperature and a stable ceramic structure, their high-temperature operating conditions can rather act as a disadvantage, causing problems such as thermal degradation of ceramic materials, sealing issues, and rapid oxidation of metal materials used as interconnectors. To solve these problems, research on Intermediate Temperature-operating Solid Oxide Fuel Cells (IT-SOFC) that feature lowered operating temperature in the intermediate temperature range (500–750°C) has been actively conducted. However, the main obstacle to this lowered-temperature-range SOFCs is the decreased electrochemical activity of the two electrodes (cathode and anode), as well as lowered ionic conductivity and increased ohmic resistance of the electrolyte. Significantly, the major source of voltage loss in IT-SOFCs is the cathode materials. Therefore, to achieve high power densities at the reduced temperatures, it is necessary to develop cathode materials that show excellent electrochemical reaction and enhanced/advanced low polarization resistance (Minh, 1993; Tu et al., 1997).

Recently, to replace existing IT-SOFC cathode materials, many research groups have investigated new concept-based cathode materials with a chemical composition of layered perovskite, because this structure yields superior oxide ionic diffusivity, excellent oxygen surface exchange coefficients, high oxygen transport properties, and higher electronic conductivity (Kim et al., 2010; Joung et al., 2013; Zhang et al., 2016). In this new perovskite structure (A^/^A^//^B_2_O_5+d_ chemical composition), various lanthanide atoms such as La, Pr, Nd, and Sm can be substituted into the A^/^-site; Ba can be replaced in the A^//^-site; and transition metal ions of Co, Mn, and Fe can be located at the B-site.

The first application of a layered perovskite structure as cathode of SOFC (Zhou et al., 1993) showed that a difference in coordination number occurs due to the difference in ion radius between the A^/^ and A^//^ ions; thus, oxygen vacancies are formed, yielding excellent electrochemical properties (Akahoshi and Ueda, 2001; Frontera et al., 2003; Kim et al., 2007, 2010; Kim and Manthiram, 2015). The layered perovskite structure into which elemental Co was substituted on the B-site is comprised of a stacked structure with [CoO_2_]-[BaO]-[CoO_2_]-[LnO_6_] on the c-axis.

In the [LnO_6_] layer, there is a large difference between the ionic radius of Ln and the ionic radius of Ba^2+^, resulting in a pyramid structure of CO_5_ and an octahedral structure of CO_6_. Finally, oxygen vacancies are generated in the [LnO_6_] layer due to this structural feature. Accordingly, structure is known to exhibit higher electrochemical properties because it has excellent oxygen mobility properties and at the same time shows excellent surface diffusion properties. For example, according to Ding et al., who investigated the composition of PrBa_0.5_Sr_0.5_Co_2_O_5+d_, this composition exhibited remarkable properties in the 500–700°C temperature range and showed an area specific resistance (ASR) value of 0.23 Ω·cm^2^ at 650°C (Ding and Xue, 2010). In additional, the SmBaCo_2_O_5+d_ layered perovskite had an ASR of 0.13 Ω·cm^2^ at 650°C (Kim et al., 2009). However, the thermal shock and long-term stability of the Co substituted layered perovskite at high temperatures have been questioned due to the high thermal expansion coefficient (TEC) (Tietz, 1999; Pelosato et al., 2015).

Strategies to develop not only excellent cathode materials for IT-SOFCs by substituting various materials into layered perovskite, but also non-stoichiometric cathode materials synthesized by removing individual elements of layered perovskite are being pursued (Kostogloudis and Ftikos, 1999; Wang et al., 2014; Sun et al., 2016; Yi et al., 2016). Based on the composition PrBaCo_2_O_5+d_, PrBa_0.92_Co_2_O_5+d_, in which 0.08 mol% of Ba was removed from the A^//^-site, showed better electrochemical properties than the composition PrBaCo_2_O_5+d_. In particular, PBa_0.92_CO had an excellent ASR value of 0.166 Ω·cm^2^ at 600°C (Idrees et al., 2019). It can be seen from this result that the electrochemical properties were enhanced in the non-stoichiometric composition in which the A^//^-site is removed stepwise from the composition of layered perovskite LnBaCo_2_O_5+d_.

In the case of the composition of PrBaCo_2_O_5+d_ with partially removed A^/^-site, not A^//^-site, the concentration of oxygen vacancies inside the crystal structure increased as the Pr-deficiency level increased. Especially, the Pr_0.95_BaCo_2_O_5+d_ composition showed ASR values of 0.113, 0.054, and 0.028 Ω·cm^2^ at 600, 650, and 700°C (Jiang et al., 2014). As another example, the oxygen content of PrBaCo_2_O_5+d_ was 5.74 and the value of Pr_0.92_BaCo_2_O_5+d_ was reduced to 5.62 when the amount of Pr removed increased. In addition, Pr_0.92_BaCo_2_O_5+d_ was found to have excellent ASR values of 0.081 and 0.041 Ω·cm^2^ at 700 and 750°C (Zhang et al., 2016). Therefore, in the case of the non-stoichiometric composition of the layered perovskite, excellent electrochemical properties can be obtained when the lanthanide element is controlled. In summary, oxygen vacancies in the lattice of layered perovskite can be activated through non-stoichiometry of the A^/^-site, thereby promoting the ORR reaction.

The goal of this research is to investigate the phase synthesis characteristics of Sm_1−x_BaCo_2_O_5+d_ (*x* = 0, 0.01, 0.02, 0.03, 0.04, 0.05, 0.10, and 0.15) showing non-stoichiometric compositions in which samarium (Sm) elements were stepwise removed at the A^/^-site of a SmBaCo_2_O_5+d_ layered perovskite by using solid state reaction. Significantly, the electrochemical properties of Sm_1−x_BaCo_2_O_5+d_ oxide systems were also investigated to apply this material as cathode material for direct application to IT-SOFCs.

## Experimental Section

### Synthesis

Sm_1−x_BaCo_2_O_5+d_ (*x* = 0, 0.01, 0.02, 0.03, 0.04, 0.05, 0.10, and 0.15) oxide systems having layered perovskite structure were synthesized using Solid State Reaction (SSR). The starting materials were Sm_2_O_3_ (smarium oxide, Alfa Aesar, 99.9%), BaCO_3_ (barium carbonate, Samchun, 99.0%), and Co_3_O_4_ (cobalt oxide, High Purity Chemicals, 99.9%); an electronic balance (WBA-320) was use for accurate weighing to the third decimal place.

In this study, two calcinations were performed for more accurate synthesis of single phase. The weighed materials were mixed and calcined for 6 h at 1,000°C in air. After the first calcination into uniform and small particles, wet ball milling was performed at room temperature for about 1 day to make the composite powder. The powder after ball milling was again calcined at a rate of 5°C/min in air, and secondary calcination was performed at 1,100°C for 8 h. Resulting materials after the two calcinations were pulverized to form fine powders. The compositions and abbreviations are summarized in Table 1.

**Table 1:** Abbreviations of Sm_1−x_BaCo_2_O_5+d_ (*x* = 0, 0.01, 0.02, 0.03, 0.04, 0.05, 0.10, and 0.15) compositions.

**Composition**	**Abbreviation**
SmBaCo_2_O_5+d_	SBCO_1
Sm_0.99_BaCo_2_O_5+d_	SBCO_0.99
Sm_0.98_BaCo_2_O_5+d_	SBCO_0.98
Sm_0.97_BaCo_2_O_5+d_	SBCO_0.97
Sm_0.96_BaCo_2_O_5+d_	SBCO_0.96
Sm_0.95_BaCo_2_O_5+d_	SBCO_0.95
Sm_0.90_BaCo_2_O_5+d_	SBCO_0.90
Sm_0.85_BaCo_2_O_5+d_	SBCO_0.85

### Sample Preparation

Ce_0.9_Gd_0.1_O_2−d_ (CGO91, Solvay) powders were used as the electrolyte of symmetrical half cells (Sm_1−x_BaCo_2_O_5+d_/CGO91/Sm_1−x_BaCo_2_O_5+d_). After weighing 2.5 g of CGO91, it was put into a metal mold and compression molding was performed by applying a force of 2 × 10^3^ kg/m^2^. After that, sintering was performed at 1,450°C for 6 h in air. Cathode ink made using a-terpieal(Kanto Chemical), Butvar(Sigma Aldrich), and acetone with cathode powder; composite ink was also prepared by mixing electrolyte powder and cathode material at a weight ratio of 50:50. Both sides of the sintered electrolyte support were printed' with the prepared cathode ink, and the printed cell was sintered at 1,000°C for 1 h in the air condition. After the sintering, surface area of both sides of cathode was measured and found to be 0.785 cm^2^. To confirm the electrical conductivity characteristics of the composites as identified through XRD analysis, a sample was prepared and the electrical conductivity was measured. After making a bar-type sample with dimensions of ~2.8 × 5.5 × 23 mm through compression molding, sample was sintered at 1,100°C for 3 h in air.

### Characterizations

To understand the crystal structure of the cathode powder synthesized by SSR method, X-ray diffraction analysis was used. The experimental equipment was an X-ray Diffractometer (Model: SmartLab/Rigaku), operated using a Cu Kα filter under conditions of 9 kW, 45 kV, and 200 mA. A range of 10–140° (2θ) was employed with steps of 0.02°. Microstructural properties of Sm_1−x_BaCo_2_O_5+d_ (*x* = 0, 0.01, 0.02, 0.03, 0.04, 0.05, 0.10, and 0.15) oxide systems were examined using a field-emission scanning electron microscope (FESEM/Hitachi SU5000). To measure the electrical conductivity, a bar-type sample was prepared and the electrical conductivity was measured through the DC 4-probe method. A Keithley 2400 Source meter was used as measuring device; after connecting the sample with Pt-wire, the electrical conductivity was measured by increasing the temperature in steps of 50°C in a temperature range of 50–900°C. Additionally, the current range was measured from 0 to 1A through steps of 0.05A, and the limit of voltage was set to 20V.

Impedance measurement was performed to confirm the electrochemical properties of the synthesized Sm_1−x_BaCo_2_O_5+d_ (*x* = 0, 0.01, 0.02, 0.03, 0.04, 0.05, 0.10, and 0.15) oxide systems. According to existing literature, SmBaCo_2_O_5+d_ does not react with Ce_0.9_Gd_0.1_O_2−d_ (CGO91), so there is no problem to use it with CGO91 (Kim et al., 2009). Therefore, the symmetrical half cell (SBCO_X/CGO91/SBCO_X) after the sintering was connected using Pt mesh, and the impedance was measured in a temperature range of 500–750°C in an air atmosphere. Frequency range was 0.05 Hz to 2.5 MHz.

The area specific resistance (ASR) of the synthesized composition was calculated using the impedance results obtained through electrochemical analysis. Using the impedance results with ohmic resistance removed, ASR was calculated according to the difference between the low intercept and high intercept of the real X-axis. Impedance was measured using a multi-channel electrochemical analyzer (Model nStat, HS Technologies).

## Results and Discussion

**Figure 1:**
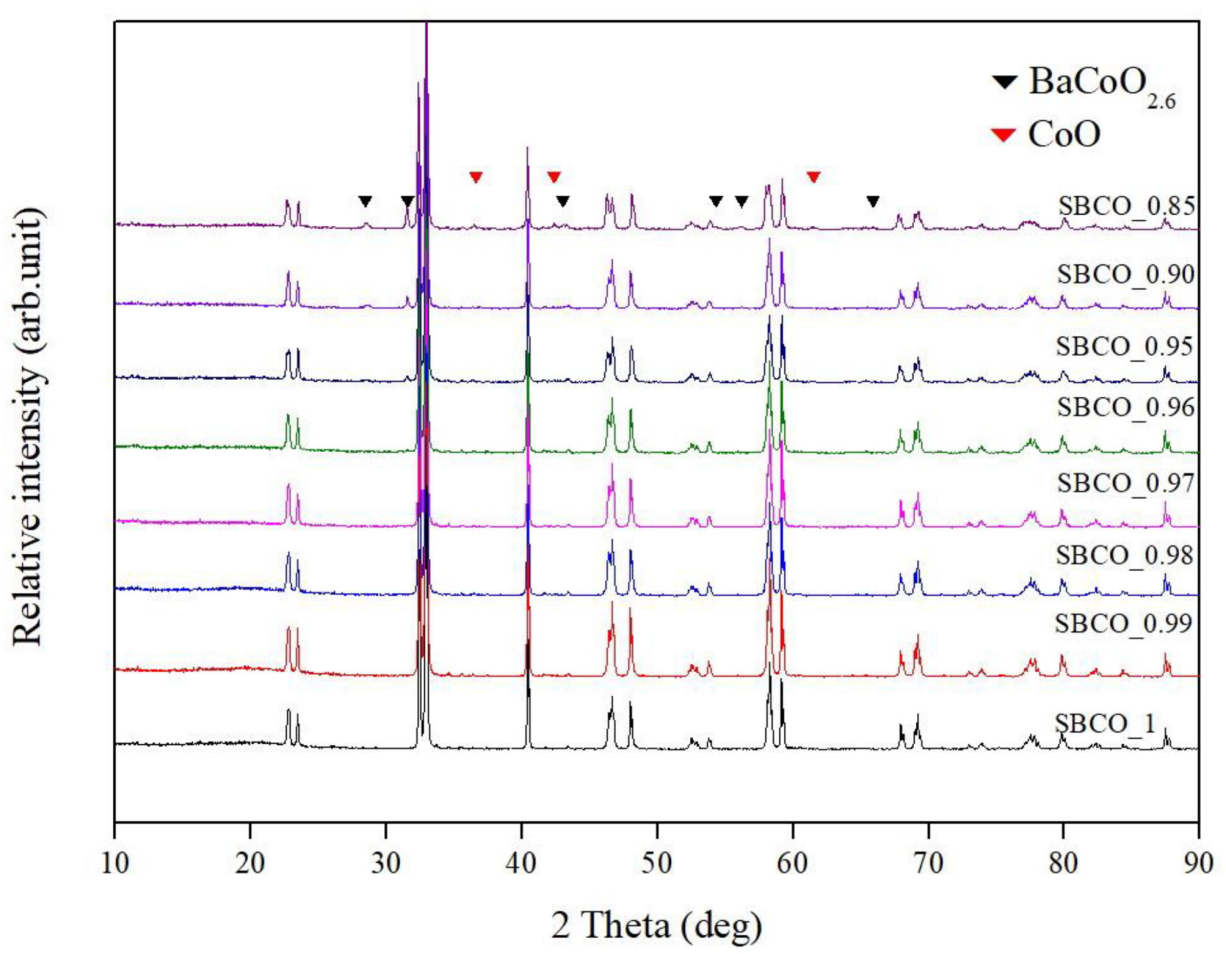
X-ray diffraction (XRD) patterns of Sm_1−x_BaCo_2_O_5+d_ (*x* = 0, 0.01, 0.02, 0.03, 0.04, 0.05, 0.10, and 0.15) oxide systems calcined at 1,100°C for 8 h.

Figure 1 summarizes the XRD results of Sm_1−x_BaCo_2_O_5+d_ (*x* = 0, 0.01, 0.02, 0.03, 0.04, 0.05, 0.10, and 0.15) calcined at 1,100°C for 8 h to investigate the synthesis characteristics of these oxide systems with respect to the non-stoichiometric compositions. In addition, indicators for BaCoO_2.6_ phase (▼) and CoO (▼) were added to all XRD results. The characteristic XRD patterns discovered in the vicinity of 23.4°, 33.0^o^, 40.4^o^, 46.7^o^, 59.1^o^, 69.2^o^, 78.1^o^) indicate that the Sm_1−x_BaCo_2_O_5+d_ oxide systems were synthesized as a single phase from the stoichiometric SmBaCo_2_O_5+d_ (SBCO) composition in which Sm was not removed and the non-stoichiometric composition in which 0.01, 0.02, 0.03, and 0.04 mol% of Sm (SBCO_0.99, SBCO_0.98, SBCO_0.97, and SBCO_0.96) were partially removed. However, when Sm was removed at 0.05 mol% or more, BaCoO_2.6_ phase appeared (SBCO_0.95, SBCO_0.90). Especially, when Sm was removed 0.15 mol%, CoO phase was additionally identified (SBCO_0.85).

Supplementary Figure 1 summarizes the SEM images of the Sm_1−x_BaCo_2_O_5+d_ (*x* = 0, 0.01, 0.05, and 0.10) oxide systems. Overall particle size decreases from (A) to (C), which results in porous microstructures as the non-stoichiometry composition increases. Especially, the particular microstructural characteristics can be seen in Supplementary Figures 1C,D. For example, in Supplementary Figure 1D, in which a part of SBCO_0.95 is magnified, white crystals can be seen protruding like arrowheads on the cathode surface. Particle size of arrowheads is very small, so it was impossible to carry out EDS (Energy-Dispersive x-ray Spectroscopy) analysis. However, as mentioned in the XRD results, it is known that the white arrowheads are formed by crystal structure of BaCoO_2.6_. In other words, it can be thought that a small amount of secondary phase particles was generated on the cathode surface. For the SBCO_0.90 composition, which further increased the non-stoichiometry, as can be seen in Supplementary Figures 1E,F, particles like pebbles can be seen attached to the cathode surface. It is found that the particle size increased as the concentration of BaCoO_2.6_ increased via the increase of the non-stoichiometry.

**Figure 2:**
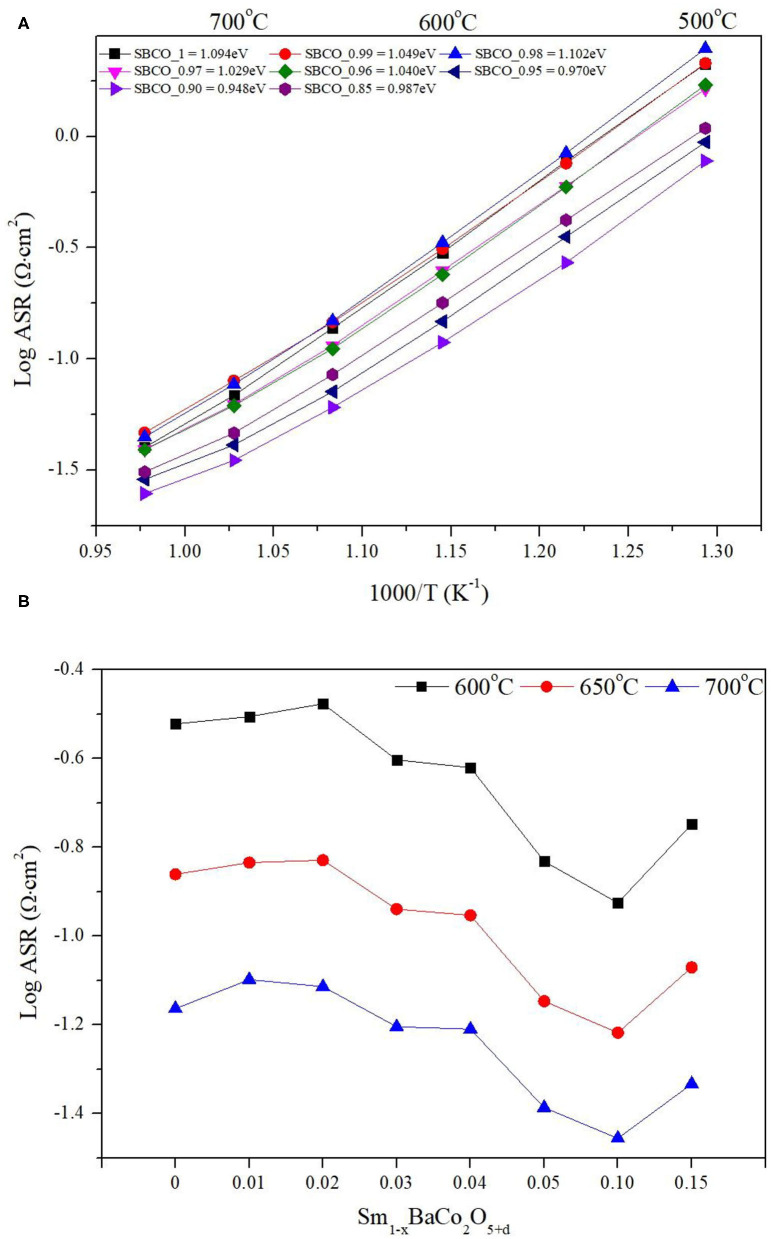
Area specific resistance (ASR) results of **(A)** Sm_1−x_BaCo_2_O_5+d_ oxide systems with respect to temperature, and **(B)** ASR dependence of cathodic polarization with respect to compositions at 600, 650, and 700°C.

Figures 2A,B show that the overall ASR value decreases as the tendency toward non-stoichiometric composition increases (= the removal amount of Sm increases). For example, the ASRs of SBCO_1, SBCO_0.95, and SBCO_0.90 were about 0.301, 0.147, and 0.119 Ω·cm^2^ at 600°C. These ASR values are closely related to the composition because the lowest ASR value was observed for the SBCO_0.90 composition. However, in the composition of SBCO_0.85 with an increased non-stoichiometric condition, the ASR value measured at 600°C was 0.179 Ω·cm^2^, which is relatively higher than the ASR values of SBCO_0.95 and SBCO_0.90 measured at 600°C. That is, from the non-stoichiometric compositions of Sm_1−x_BaCo_2_O_5+d_, the characteristics of the percolation composition showing the lowest ASR value in the chemical composition of Sm_0.90_BaCo_2_O_5+d_ can be found. This tendency can also be confirmed in the layered perovskite composition of LnBaCo_2_O_5+d_ in which Pr was substituted into the A^/^-site of the layered perovskite (Zhang et al., 2016; Idrees et al., 2019).

For example, according to the literature on ASR characteristics caused by non-stoichiometric composition of Pr_1−x_BaCo_2_O_5+d_ (*x* = 0–0.1), PrBaCo_2_O_5+d_ (PBCO) and P_0.92_BaCo_2_O_5+d_ (PBCO_0.92) exhibit ASR values of about 0.097 and 0.081 Ω·cm^2^ at 700°C (Zhang et al., 2016). In the case of electrochemical properties related to the non-stoichiometric composition that reduce the amount of Pr in Pr_1−x_BaCo_2_O_5+d_ oxide systems, when the degree of non-stoichiometry of the A^/^-site increased, the ASR tended to decrease; at the same time, the percolation composition was also exhibited.

As the degree of non-stoichiometry of the A^/^-site increases in the Sm_1−x_BaCo_2_O_5+d_ oxide systems, the value of the activation energy tends to decrease. This trend can also be confirmed in works in the literature that have studied the electrochemical properties of similar layered perovskite structure composition in which the A^/^-site is partially removed (Jiang et al., 2014; Yi et al., 2016). As a result of calculating the activation energy values, as shown in Figure 2A, Sm_0.98_BaCo_2_O_5+d_ and Sm_0.96_BaCo_2_O_5+d_ compositions have slightly increased activation energy values, but overall activation energy showed a tendency to decrease. For example, the activation energy of SmBaCo_2_O_5+d_ was 1.094 eV. As degree of non-stoichiometry of A^/^-site increases, the activation energy decreases. The values of Sm_0.95_BaCo_2_O_5+d_ and Sm_0.90_BaCo_2_O_5+d_ were calculated and found to be 0.970 and 0.948 eV. In addition, the value (0.987eV) of Sm_0.85_BaCo_2_O_5+d_ increased slightly compared to that of Sm_0.90_BaCo_2_O_5+d_. When comparing the activation values of the Sm_1−x_BaCo_2_O_5+d_ oxide systems synthesized in this experiment with those of recently reported cathode materials showing excellent electrochemical reaction, it can be determined that Sm_1−x_BaCo_2_O_5+d_ oxide systems have relatively lower activation energy (Clematis et al., 2019; Anbo et al., 2020). In general, the activation energy indicates the strength of the energy barrier that must be overcome when oxygen ions move through the oxygen vacancies (Kaur and Singh, 2020).

For example, as mentioned above, Jiang et al. reported on the compositions of PBCO and P_0.95_BCO; these compositions showed activation energy values of 1.19 and 0.98 eV (Jiang et al., 2014). This activation energy phenomenon was caused by the increase of concentration of pathways through which oxygen ions could move because the deficiency of Pr in A^/^-sites resulted in many more oxygen vacancies in the PrO layer. The activation energy value was an 0.948 eV, excellent even when compared with those of other compositions of layered perovskite (Jiang et al., 2014; Yi et al., 2016). In addition, as shown in Figure 2B, Sm_0.90_BaCo_2_O_5+d_ had the lowest ASR values in this experiment, showing values of 0.119, 0.061, and 0.035 Ω·cm^2^ at 600, 650, and 750°C, respectively.

**Figure 3:**
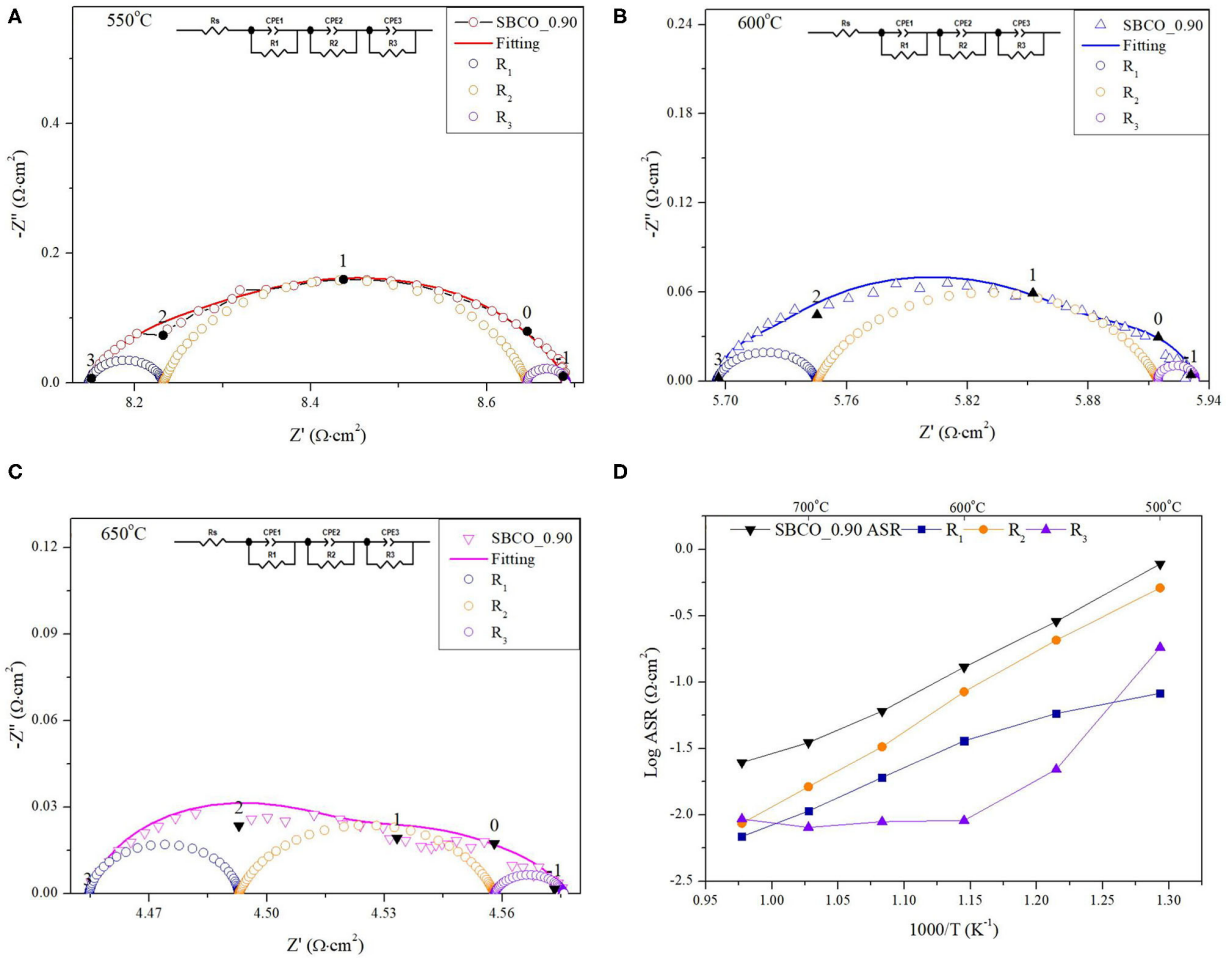
Impedance plots of Sm_0.90_BaCo_2_O_5+d_ measured at **(A)** 550°C, **(B)** 600°C, and **(C)** 650°C with equivalent circuit in inset. **(D)** shows ASR calculated from R_1_ (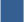), R_2_ (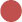), and R_3_ (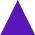) of equivalent circuit; total ASRs are shown as (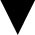).

The results of an equivalent circuit of SBCO_0.90 are given in Table 2 and Figure 3 to confirm the rate determining step (RDS). In Table 2, the impedance results measured at 600°C using the Sm_1−x_BaCo_2_O_5+d_ oxide systems were divided into R_1_, R_2_, and R_3_.

**Table 2:** ASR results of symmetrical half cells comprised of Sm_1−x_BaCo_2_O_5+d_/CGO 91/Sm_1−x_BaCo_2_O_5+d_ (*x* = 0, 0.01, 0.02, 0.03, 0.04, 0.05, 0.10, and 0.15) oxide systems measured at 600°C.

**Composition**	**R_**1**_(2.5 MHz−100 Hz) Ω·cm^**2**^**	**R_**2**_(100 Hz–1 Hz) Ω·cm^**2**^**	**R_**3**_(1 Hz–0.05 Hz) Ω·cm^**2**^**
SBCO_1	0.0741	0.2162	0.0104
SBCO_0.99	0.0550	0.2405	0.0167
SBCO_0.98	0.0558	0.2628	0.0152
SBCO_0.97	0.0550	0.1826	0.0118
SBCO_0.96	0.0559	0.1702	0.0135
SBCO_0.95	0.0279	0.1112	0.0082
SBCO_0.90	0.0250	0.0847	0.0091
SBCO_0.85	0.0317	0.1355	0.0115

In general, the impedance results of SOFC cathode materials can be classified by the frequency range (Murray et al., 1998). In equivalent circuits, the resistance from the high frequency (HF, 10^3^ Hz~) is directly related with the charge transfer (R_1_) when oxygen ions move across the interface between the cathode and the electrolyte. The resistance from the middle frequency (MF, 10^2^~1 Hz) range is attributed to the resistance (R_2_) that occurs when oxygen ions move inside the bulk of the cathode. The lower frequency (LF, below 1 Hz) arc is ascribed to oxygen dissociation and bulk or surface oxygen diffusion process (R_3_). Therefore, ASR results were analyzed by subdividing them into R_1_, R_2_, and R_3_.

In Figures 3A–C, the results for an equivalent circuit consisting of R_1_, R_2_, and R_3_ and the impedance results measured directly for the SBCO_0.90 composition at 550, 600, and 650°C are summarized. Figure 3D presents the impedance results of SBCO_0.90 measured in the temperature range of 500~750°C, as well as the values R_1_, R_2_, and R_3_ separating these results, obtained using an equivalent circuit.

As can be seen from Figures 3A–C, R_2_ takes up the largest proportion of the total resistance. These results are also summarized in Table 2. In addition, all the compositions of Sm_1−x_BaCo_2_O_5+d_ (*x* = 0, 0.01, 0.02, 0.03, 0.04, 0.05, 0.10, and 0.15) applied in this experiment showed the same results. For example, R_2_ of SBCO_1 was calculated and found to be 0.2162 Ω·cm^2^, which is about 72% of the total ASR; however, R_1_ (0.074 Ω·cm^2^) and R_3_ (0.0104 Ω·cm^2^) occupied only 28% of the total resistance. This trend does not change with increasing degree of non-stoichiometry of compositions and the R_2_ values of SBCO_0.95 and SBCO_0.90 are 0.1112 and 0.0847 Ω·cm^2^, with proportions of about 75 and 71% of the total resistance. Therefore, it can be determined that the RDS of Sm_1−x_BaCo_2_O_5+d_ (*x* = 0, 0.01, 0.02, 0.03, 0.04, 0.05, 0.10, and 0.15) oxide systems tested is R_2_, which is the resistance when oxygen ions move in the cathode bulk. In addition, in Table 2, all R_1_, R_2_, and R_3_ ASR values decreased as the non-stoichiometry compositions increased. Especially for the SBCO_0.95 composition, ASR decreases rapidly. The reason for the rapid decrease in ASR results for SBCO_0.95 is the effect of BaCoO_2.6_ phase, as described in the above XRD and SEM results. In the case of the SBCO_0.90 composition, which showed increased concentration of BaCoO_2.6_, the ASR value was lower than that of SBCO_0.95.

According to Qi et al., who studied the composition of the perovskite structure BaCo_0.6_Zr_0.4_O_3−δ_ (BZC-BC) in nanocomposite form, advanced electrochemical properties were reported when a secondary phase of BaCo_0.96_Zr_0.04_O_3−δ_ transformed into BaZr_0.82_Co_0.18_O_3−δ_ main phase. For example, single-phase BaZr_0.80_Co_0.20_O_3−δ_ (BZC2) showed a power density of 33 mW·cm^−2^ at 750°C. However, the BCZ-BC oxide system containing the secondary phase (BaCo_0.96_Zr_0.04_O_3−δ_) showed a value of about 1,530 mW·cm^−2^, an increase of 46 times compared to the case of using single phase at the same temperature; this implies that the composition BZC-BC including secondary phase exhibited excellent properties. In addition, in the case of multi-phase cathode materials mixed with various compositions containing Ba and Co based oxide, it was reported that BaCoO_3−x_ generates a large number of oxygen vacancies that promote the oxygen reduction reaction (ORR) by assisting the movement of reduced oxygen ions (Qi et al., 2020). Therefore, the composition of SBCO_0.90 applied in this experiment, which has the lowest ASR characteristics, is affected by activated ORR caused by BaCoO_2.6_.

**Figure 4:**
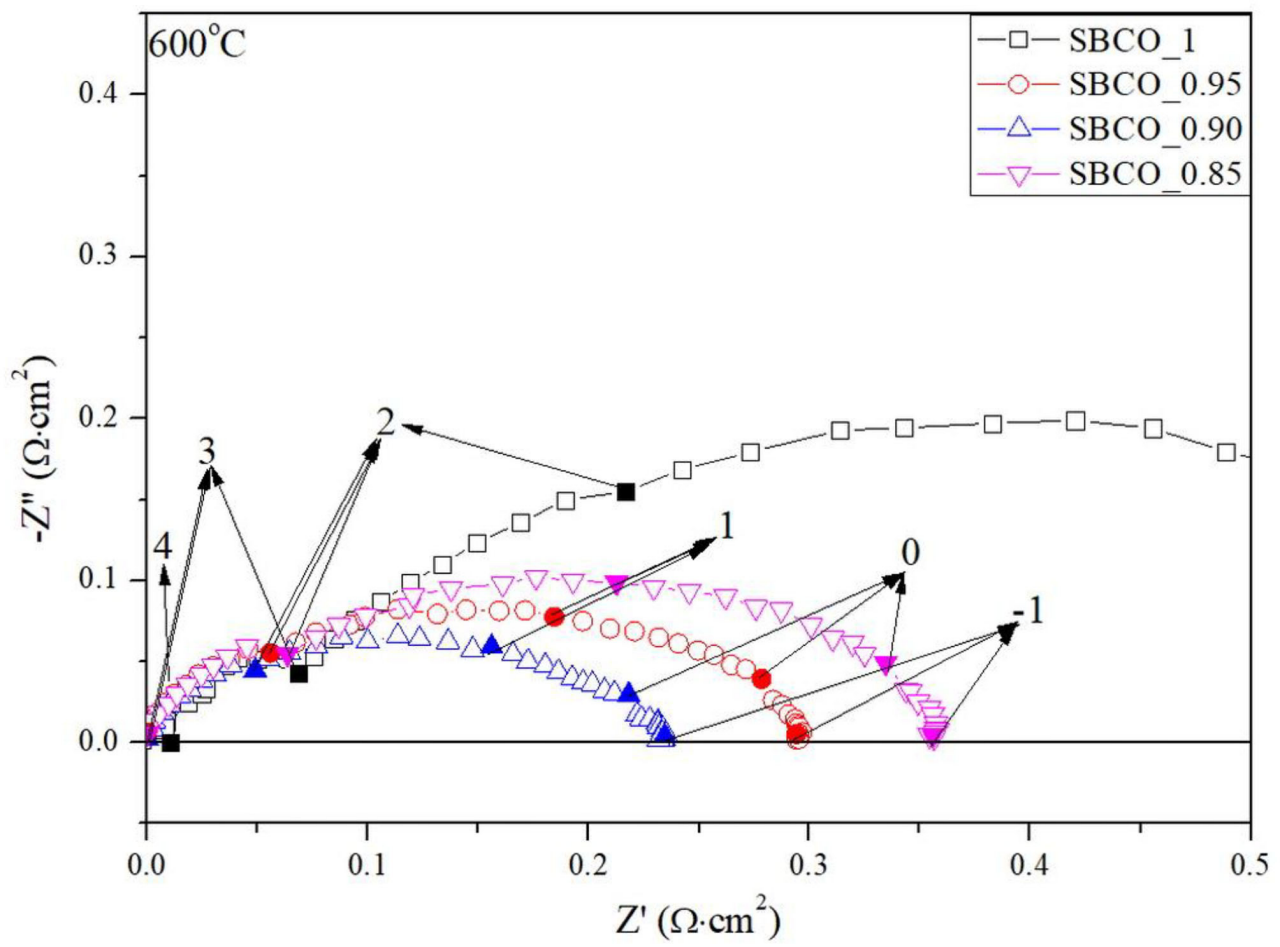
Impedance results of Sm_1−x_BaCo_2_O_5+d_ (*x* = 0, 0.05, 0.10, and 0.15) measured at 600°C. For exact comparisons of ASRs, ohmic resistances were removed in these results; inset numbers denote logarithm of measuring frequency.

Figure 4 summarizes the impedance results of the Sm_1−x_BaCo_2_O_5+d_ (*x* = 0, 0.05, 0.10, and 0.15) oxide systems measured at 600°C. ASR values decrease as the effect of degree of non-stoichiometry composition increased. However, in the SBCO_0.85 composition, the R_2_ value increased sharply and the total ASR increased. From these results, it can be determined that the CoO phase found in the composition of SBCO_0.85, shown in Figure 1, somewhat limits the movement of oxygen ions in the bulk state of the cathode.

**Figure 5:**
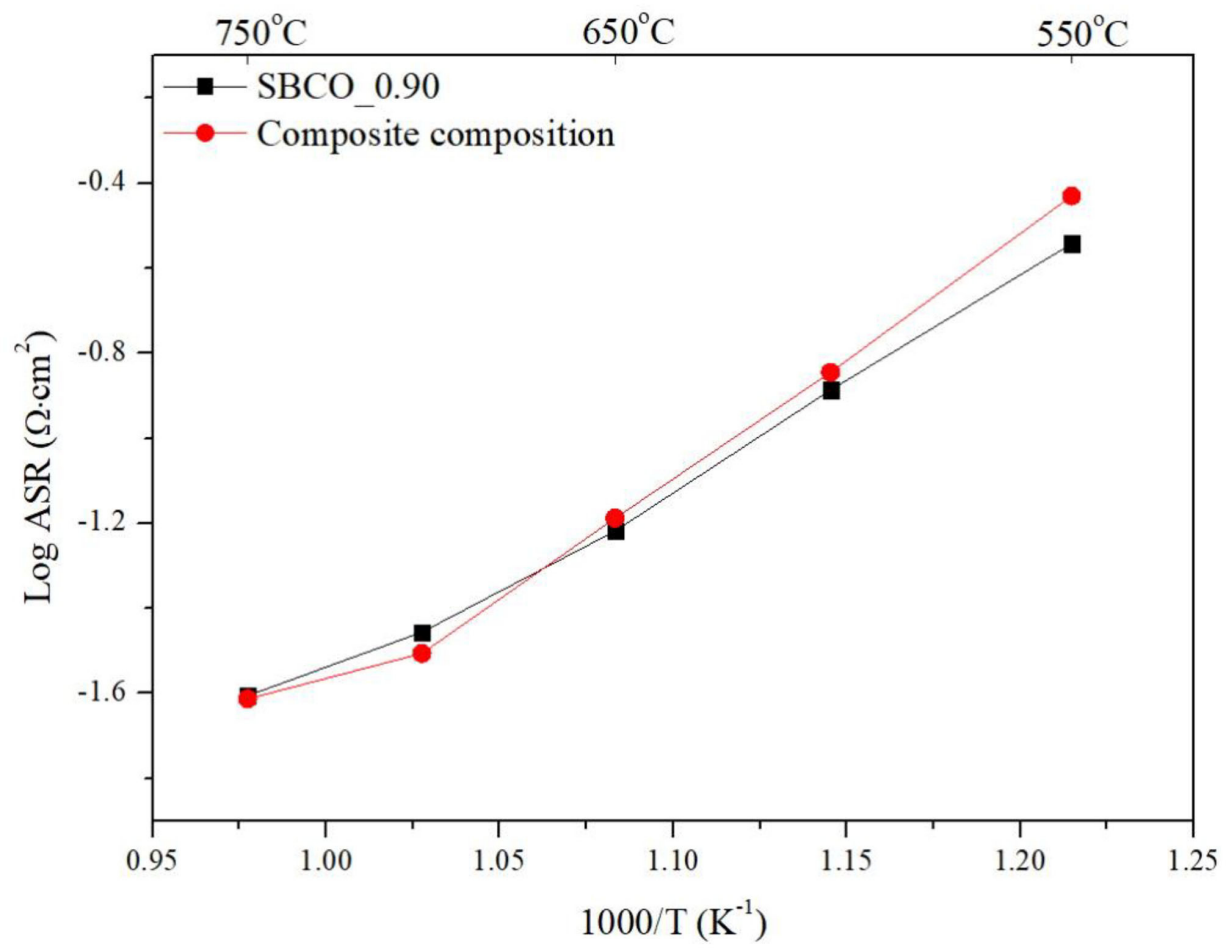
Area specific resistances (ASRs) of SBCO_90 and composite cathode on dense CGO91 electrolyte, measured from 550 to 750°C.

Additionally, electrochemical analysis was carried out using composite cathodes prepared by mixing CGO91 powder and SBCO_0.90. For the fabrication of the composite cathodes, the synthesized SBCO_0.90 was mixed with CGO91 at a weight ratio of 1:1. The summarized ASR results of the composite cathodes are given in Figure 5 and Table 3. As can be seen in Figure 5, the two cathode materials of single phase SBCO_0.90 and composite phase exhibited similar ASR values over the entire measured temperature range. For example, the composite cathode showed ASR values of 0.031 and 0.024 Ω·cm^2^ at 700 and 750°C; it showed a slight difference of only 0.004 Ω·cm^2^ compared to the ASR value of SBCO_0.90. In the general case of composite cathodes, ASRs measured from the composite cathode tend to decrease. However, a large amount of CGO91 could rather interfere with the movement of oxygen ions in the bulk and on the surface of the cathode, and so composite did not significantly affect the reduction of resistance of the composite cathode applied in this experiment.

**Table 3:** ASR comparisons of single phase cathode SBCO_0.90 and composite cathode measured from 550 to 750°C.

**Composition**	**550**	**600**	**650**	**700**	**750**	**T(°C)**
SBCO_0.90	0.271	0.119	0.061	0.035	0.025	Ω·cm^2^
Composite	0.349	0.139	0.056	0.031	0.024	Ω·cm^2^

From these results, it can be confirmed that the effect of the composite cathode is not significant for the non-stoichiometric composition state in which the quantitative composition of the A^/^-site of the layered perovskite changes. However, the thermal expansion problem of the SOFC cathode operating at high temperature range can be solved when the composite cathode concept is applied. In other words, thermal stability can be secured by lowering the thermal expansion coefficient (TEC). Co-based materials have high TEC of 20–25 × 10^−6^K^−1^ when SOFC is operated in high temperature operating temperature range; however, CGO91 has a relatively low TEC of 12–13.1 × 10^−6^K^−1^ (Kim et al., 2009). Therefore, the high TEC value generated from the cathode material substituted with Co can lower the coefficient of thermal expansion through composite cathodes.

Kim et al., who synthesized the composition of SmBaCo_2_O_5+d_ through in a composite with CGO91 at an ~50:50 ratio, reported that the TEC of 20.2 × 10^−6^K^−1^ decreased by about 66% to 13.3 × 10^−6^K^−1^ (Kim et al., 2009). By mixing the electrolyte materials and cathode materials, the thermal stability increases while the TECs rapidly decrease. Therefore, it is suitable to use a composite cathode with excellent electrochemical properties and relatively lower TEC when applying single phase SBCO_0.90 at a relatively high temperature (650°C~). However, it is desirable to use a single phase cathode when using at a relatively lower temperature range.

The ASRs of Sm_1−x_BaCo_2_O_5+d_ (*x* = 0, 0.01, 0.02, 0.03, 0.04, 0.05, 0.10, and 0.15) applied in this study were 0.083, 0.095, 0.088, 0.074, 0.075, 0.041, 0.035, and 0.045 Ω·cm^2^, respectively, at 700°C. All compositions can be sufficiently used as cathode materials at 700°C because these ASR results are all 0.15 Ω·Cm^2^ or less (Steele, 1996). The ASRs of SBCO_0.97, SBCO_0.96, SBCO_0.95, and SBCO_0.85 are 0.130, 0.129, 0.072, and 0.085 Ω·cm^2^ at 650°C. Especially, the SBCO_0.90 composition has an ASR of 0.130 Ω·cm^2^, and can thus be used as a cathode material at a relatively low temperature of 600°C.

**Figure 6:**
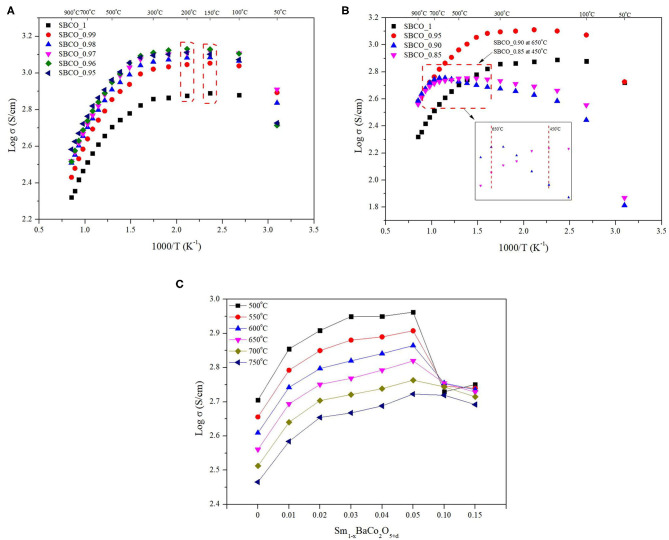
Electrical conductivity results of Sm_1−x_BaCo_2_O_5+d_ oxide systems cathode materials for each condition. **(A)** Electrical conductivity results of Sm_1−x_BaCo_2_O_5+d_ (*x* = 0, 0.01, 0.02, 0.03, 0.04, and 0.05) from 50 to 900°C in air. **(B)** Electrical conductivity results of Sm_1−x_BaCo_2_O_5+d_ (*X* = 0, 0.05, 0.10, and 0.15) 50–900°C in air. **(C)** Electrical conductivity results of Sm_1−x_BaCo_2_O_5+d_ (*x* = 0, 0.01, 0.02, 0.03, 0.04, 0.05, 0.10, and 0.15) from 500 to 750°C in air.

The electrical conductivity results of the Sm_1−x_BaCo_2_O_5+d_ (*x* = 0, 0.01, 0.02, 0.03, 0.04, and 0.05) oxide systems are summarized in Figure 6A. According to the literature related to the electrical conductivity behavior of SmBaCo_2_O_5+d_ (SBCO, in this study SBCO_1), SBCO showed a maximum electrical conductivity value (570 S/cm) in the range of 200–250°C; then, the electrical conductivity started to decrease from 250°C, indicating Metal Insulator Transition (MIT) behavior (Kim et al., 2009). The electrical conductivity tendency of MIT in the LnBaCo_2_O_5+d_ system is influenced by various factors, such as small degree of polaron hopping and oxygen content (Maignan et al., 1999; Moon et al., 2001).

However, in Figures 6A,B, it can be seen that the temperature at which the change in electrical conductivity occurs is directly related to the non-stoichiometric composition. For the compositions of SBCO_1, SBCO_0.99, SBCO_0.98, and SBCO_0.97, which are relatively less influenced by the non-stoichiometric composition, the electrical conductivity value increases from 50°C and then begins to decrease at 200°C. For the SBCO_0.96 and SBCO_0.95 compositions, which show increased influence of the non-stoichiometric composition in the A^/^-site, the electrical conductivity began to decrease at 250°C, as shown in Figure 6A. On the other hand, the electrical conductivity of SBCO_0.90 decreased from 650°C and the electrical conductivity values of SBCO_0.85 began to decrease at 450°C, as shown in Figure 6B.

From these results, it can be concluded that when the influence of the non-stoichiometric composition increases, temperature of the MIT behavior increases. The electrical conductivities of materials having non-stoichiometric compositions, namely the Sm_1−x_BaCo_2_O_5+d_ (*x* = 0, 0.01, 0.02, 0.03, 0.04, 0.05, 0.10, and 0.15) oxide systems, are summarized in Figure 6C for the intermediate temperature range (500–750°C). The values of electrical conductivity increase as the effect of non-stoichiometric composition increases (*x* ≤ 0.05). That is, the electrical conductivity increases when a relatively small amount of Sm is removed in the range of Sm_1−x_BaCo_2_O_5+d_ (*x* = 0, 0.01, 0.02, 0.03, 0.04, and 0.05). Especially the SBCO_0.95 composition exhibited electrical conductivity values of about 917 and 732 S/cm at 500 and 600°C and showed the highest electrical conductivity values among the measured compositions. However, when the non-stoichiometric composition increased from *x* = 0.05 in the Sm_1−x_BaCo_2_O_5+d_ (*x* = 0, 0.01, 0.02, 0.03, 0.04, 0.05, 0.10, and 0.15) oxide systems, the electrical conductivity value decreased. It can be concluded that this is because a large amount of BaCoO_2.6_ phase activates the conductivity of ions but inhibits the transfer of charge carriers (Chen et al., 2011; Liu et al., 2011).

Therefore, it can be seen that a percolation phenomenon that affects the electrical conductivity according to the non-stoichiometry is found in the Sm_1−x_BaCo_2_O_5+d_ (*x* = 0, 0.01, 0.02, 0.03, 0.04, 0.05, 0.10, and 0.15) oxide systems. In addition, the conductivity characteristics found in the non-stoichiometric compositions exceeding *x* = 0.04 can be determined by the influence of the secondary phase. However, the detailed influence of the BaCoO_2.6_ composition for electrical conductivity in SmBaCo_2_O_5+d_ oxide systems is not discussed now and will be reported later.

Therefore, it can be determined that as the deficiency level of A^/^-site increases, the electrical conductivity behavior conditions in this experiment changed. In addition, the lowest conductivity value in the IT range was 169 S/cm, a value exceeding 100 S/cm. This is a value that exceeds the standard that must be satisfied for material to be used as cathode for SOFC; therefore, all compositions can be suitable for use as cathode material for IT-SOFC (Tu et al., 1997). In addition, SBCO_0.95, having the highest electrical conductivity values in the IT ranges, also showed electrical conductivity values higher than those of NdBaCo_2_O_6−d_ and the compositions of LnBaCo_2_O_5+d_ (Ln = La, Pr, Nd, Sm, Gd, Y) (Kim and Manthiram, 2015; Yi et al., 2016). Especially SBCO_0.90, which shows excellent electrochemical properties, also showed a high electrical conductivity value of 500 S/cm or more in the IT range.

## Conclusion

The electrochemical properties of materials having non-stoichiometric compositions in which Sm of the A^/^-site was partially removed in SmBaCo_2_O_5+d_ oxide systems were studied for direct application of these materials as cathodes for IT-SOFC. In the case of the Sm_1−x_BaCo_2_O_5+d_ (*x* = 0, 0.01, 0.02, 0.03, 0.04, 0.05, 0.10, and 0.15) oxide systems synthesized by SSR, single phase composition was confirmed for Sm_0.96_BaCo_2_O_5+d_ (SBCO_0.96). However, secondary phase of BaCoO_2.6_ was found for compositions of Sm_0.95_BaCo_2_O_5+d_, (SBCO_0.95) in Sm_1−x_BaCo_2_O_5+d_ oxide systems. Additional phase of CoO appeared for the SBCO_0.85 composition.

All of the synthesized compositions showed excellent electrochemical properties, with ASR values <0.15 Ω·cm^2^ at 650°C. The Sm_0.90_BaCo_2_O_5+d_ (SBCO_0.90) showed the lowest ASR characteristics, with values of 0.119, 0.061, and 0.035 Ω·cm^2^ at 600, 650, and 700°C. Additionally, the ASRs of the composite cathodes prepared by mixing with an electrolyte using CGO91 and SBCO_0.90 were 0.139 and 0.056 Ω·cm^2^ at 600 and 650°C. All compositions exhibit electrical conductivity values of more than 100 S/cm over the temperature range (500–750°C) for IT-SOFC. It was also confirmed that the behavior conditions of MIT change as the non-stoichiometry composition of the A^/^-site increases. SBCO_0.90 composition, which had the best electrochemical properties, showed the maximum (568 S/cm) and minimum (387 S/cm) electrical conductivity values in the temperature range of 500–900°C.

## Data Availability Statement

The original contributions presented in the study are included in the article/Supplementary Material, further inquiries can be directed to the corresponding author/s.

## Author Contributions

CK was responsible for conducting all experiments, analyzing experimental data, and writing papers. SW teaches experimental methods and informs questions about results. KS, S-WB, HK, and WC helped to write the manuscript. KS provided additional help in thesis publication. Finally, JK coordinated the overall experimental design, direction, and schedule. In addition, he reviewed the paper and gave direction. All authors contributed to the article and approved the submitted version.

## Conflict of Interest

The authors declare that the research was conducted in the absence of any commercial or financial relationships that could be construed as a potential conflict of interest.
