# Supplementary material for: Enhanced Electrochemical Properties of Non-stoichiometric Layered Perovskites, Sm1−xBaCo2O5+d, for IT-SOFC Cathodes
Source: Front Chem. 2021 Apr 21;9:633868. doi: 10.3389/fchem.2021.633868 (PMC8097098; doi:10.3389/fchem.2021.633868)
Supplement: Supplementary file 1 [file Image_1.PDF]

Supplementary Material

Enhanced electrochemical properties of non-stoichiometric layered perovskites, $Sm_{1-x}BaCo_2O_{5+d}$, for IT-SOFC cathodes

Chan Gyu Kim[1], Sung Hun Woo[1], Kyeong Eun Song[1], Seung-Wook Baek[2] Hyunil Kang[3], Won Seok Choi[3], Jung Hyun Kim[1]*

[1] Department of Advanced Materials Science and Engineering, Hanbat National University, 125, Dongseo-Daero, Yuseong-Gu, Daejeon, 34158, Republic of Korea

[2] Interdisciplinary Materials Measurement Institute, Korea Research Institute of Standards and Science (KRISS), 267, Gajeong-Ro, Yuseong-Gu, Daejeon, 34113, Republic of Korea

[3] Department of Electrical Engineering, Hanbat National University, 125, Dongseo-Daero, Yuseong-Gu, Daejeon, 34158, Republic of Korea

* Correspondence:

Corresponding Author: Jung Hyun Kim

jhkim2011@hanbat.ac.kr, jhkim1870@gmail.com

Tel: +82-42-821-1239, Fax: +82-42-821-1592,

Department of Advanced Materials Science and Engineering, Hanbat National University, 125, Dongseo-Daero, Yuseong-Gu, Daejeon, 34158, Republic of Korea

1 Supplementary Figures and Tables

1.1 Supplementary Figures

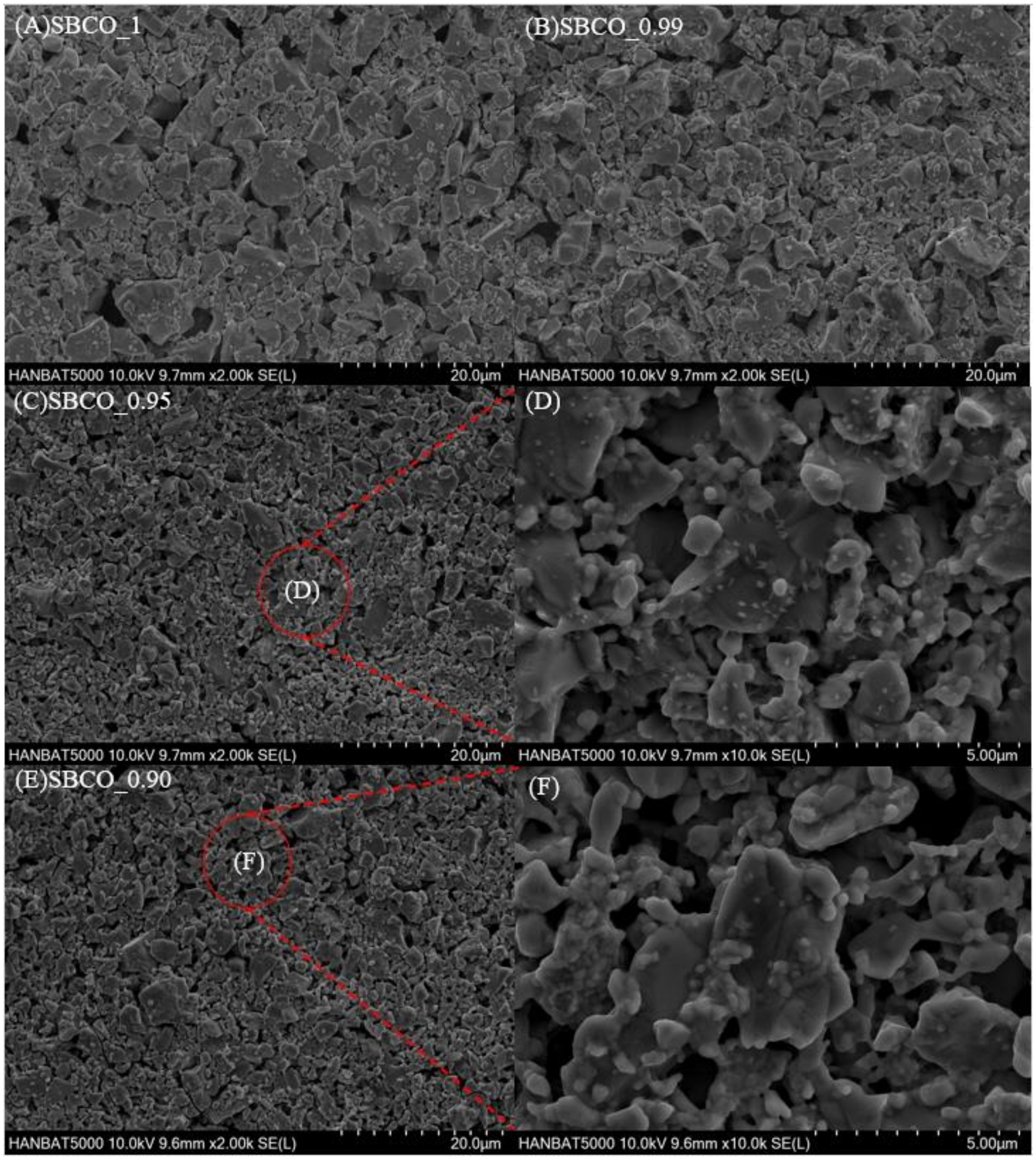

Supplementary Figure 1. Scanning electron microscopy (SEM) images of $Sm_{1-x}BaCo_2O_{5+d}$ (x=0, 0.01, 0.05, and 0.10) oxide systems. (A) SBCO_1, (B) SBCO_0.99, (C, D) SBCO_0.95, and (E, F) SBCO_0.90.
